# A Knowledge-Based Approach to Automatic Detection of Equipment Alarm Sounds in a Neonatal Intensive Care Unit Environment

**DOI:** 10.1109/JTEHM.2017.2781224

**Published:** 2017-12-22

**Authors:** Ganna Raboshchuk, Climent Nadeu, Peter Jančovič, Alex Peiró Lilja, Münevver Köküer, Blanca Muñoz Mahamud, Ana Riverola De Veciana

**Affiliations:** TALP Research CenterDepartment of Signal Theory and CommunicationsUniversitat Politècnica de Catalunya08034BarcelonaSpain; Department of Electronic, Electrical and Systems EngineeringUniversity of BirminghamBirminghamB15 2TTU.K.; NeonatologyHospital Sant Joan de Déu08950BarcelonaSpain

**Keywords:** Acoustic event detection, alarm detection, neonatal intensive care unit, sinusoid detection, non-negative matrix factorization, neural networks

## Abstract

A large number of alarm sounds triggered by biomedical equipment occur frequently in the noisy environment of a neonatal intensive care unit (NICU) and play a key role in providing healthcare. In this paper, our work on the development of an automatic system for detection of acoustic alarms in that difficult environment is presented. Such automatic detection system is needed for the investigation of how a preterm infant reacts to auditory stimuli of the NICU environment and for an improved real-time patient monitoring. The approach presented in this paper consists of using the available knowledge about each alarm class in the design of the detection system. The information about the frequency structure is used in the feature extraction stage, and the time structure knowledge is incorporated at the post-processing stage. Several alternative methods are compared for feature extraction, modeling, and post-processing. The detection performance is evaluated with real data recorded in the NICU of the hospital, and by using both frame-level and period-level metrics. The experimental results show that the inclusion of both spectral and temporal information allows to improve the baseline detection performance by more than 60%.

## Graphical abstract

A system for automatic detection of alarm sounds that uses the knowledge about their frequency and time structure.

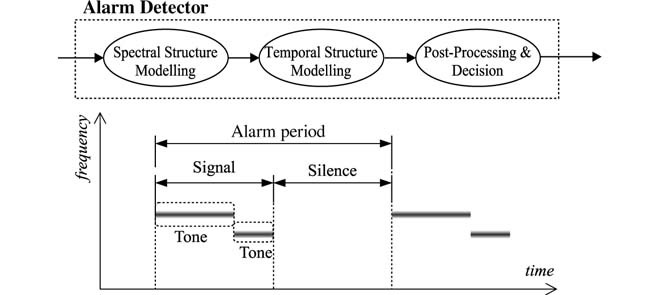

## I. Introduction

Very low birth weight preterms usually spend the first several weeks or even months of life receiving specialized care in a Neonatal Intensive Care Unit (NICU), what is crucial for their survival. In the acoustically rich environment of a typical NICU numerous sounds coming from various human activities and biomedical equipment [1], [2] often take place simultaneously and contribute to high sound pressure levels [3]. It is well known that such a noisy environment may negatively affect the growth and neurodevelopment of the premature infants [4]–[7] and is of great medical concern.

Equipment alarm sounds provide alerts about the changes in preterm infant’s condition to the medical staff and occur frequently in a NICU environment. In fact, a large number of triggered alarms are not related to any clinically relevant and/or emergency event [8], which may lead to alarm fatigue. Smart alarming systems are being designed [8], [9] to improve the alarm handling process in NICUs and reduce noise levels. Such systems use alternative alerting modalities and only allow the most critical alarms to sound. Unfortunately, in the majority of the NICUs such systems are not developed yet.

This paper presents our work on the development of an automatic system for detection of alarm sounds, which can be useful for medical application in two ways.

First, for detecting the sounds that are potentially harmful for a preterm infant due to their particular spectro-temporal structure (i.e. beats, tones and specially high frequencies). The effects of a NICU acoustic environment on a preterm infant could be revealed by the infant reactions to auditory stimuli from it, which can be investigated by relating the presence of specific sounds (i.e. sound identities and their situation in time) with the preterm physiological variables. Such investigation can complement greatly the work already reported in the literature, in which only the sound pressure level is considered (e.g. in [10]), and requires big amounts of labelled audio data, which can hardly be obtained without using automatic detection from audio signals.

Second, for assisting the medical staff in their work and facilitate the reaction to events. E.g. in [11] a sound-activated light device was implemented for alerting the staff members when the sound pressure level exceeded a predefined value. The automatic alarm detection system can be a part of a more sophisticated notification system allowing smart alarm handling algorithms, which could be designed to warn about triggering of particular alarms, to take into account their clinical relevance and urgency, etc.

The automatic alarm sound detection was previously investigated for the purposes of hearing impaired assistance and hearing support in noisy conditions [12]–[14]. To our knowledge, research on the topic was first reported in [15], where the detection of various real-world alarm sounds was addressed. In that work, two different approaches were presented: a generic model-based approach that employs features capturing the global properties of the spectrum and neural networks, and a non-model-based approach that employs sinusoid modelling and separation and exploits the specific time-frequency structure of alarms. While the model-based approach is also followed in [12], most of the posterior works adhere to the non-model-based approach. For instance, a simple signal processing based method was reported in [13], where an autocorrelation function, used to exploit the long-term periodicity of alarms, is compared to a threshold. In [14] amplitude periodicity in a specific frequency bandwidth is detected using a decision tree based on zero-crossing rate of the autocorrelation of the signal envelope. In [16] the decision is made by comparing the presence probability to a predefined threshold, where the probability is constructed based on pitch detection in specific frequency bands. Differently, in [17] the spectrogram is treated as an image and part-based models are trained in the spectro-temporal domain, providing flexibility in time and in frequency.

The non-model-based systems usually take advantage of the particular properties of alarms and their performance depends strongly on the proper choice of the decision thresholds. The model-based systems, on the other hand, require model training on a multitude of alarm samples in multiple conditions, and the amount of training data is usually an important factor.

In relation to the reported works, this work presents a combination of the model and non-model based approaches that takes advantage of both of them. The proposed detection system employs statistical modelling of the training data, but also uses the knowledge about spectral and temporal alarm characteristics. The spectral information is captured in a feature vector, which is obtained by applying either a method for detection of sinusoids (previously published in [18]) or the non-negative matrix factorization algorithm, in frequency intervals corresponding to alarm-specific frequencies. The temporal information is incorporated at the post-processing stage by aggregating the frame-level posterior probabilities, obtained from statistical modelling, along the intervals corresponding to the signal and silence segments in an alarm period, and a threshold is applied to that estimate to perform detection at the alarm period level. A non-model-based system that exploits the knowledge about the alarm characteristics in a similar manner was reported in [19]. Unlike in that work, our system is able to take into account the frequency and duration variation observed in alarms and incorporates the alarm spectral amplitude structure, which may be important for discrimination of the alarms that share some frequency components.

Starting from a basic machine learning system (baseline), which gives a very low detection performance, we introduce improvements sequentially at each of its stages and seek to obtain better detection results. Apart from the widely used frame-level metrics, we present the results using a metric that operates at the alarm period level, which is more meaningful for the medical application.

A preliminary version of this work has been reported in [20]. In comparison to that paper, here a more detailed analysis of the acoustic alarm classes is presented, different type of features and classifiers are compared, an optimised decision threshold based on the Equal Error Rate (EER) criterion is employed and a more comprehensive analysis of results is provided, including the analysis of the system performance in different Signal-to-Noise Ratio (SNR) ranges.

The rest of the paper is organized as follows. In Section II the produced database and the acoustic alarm classes are described. The general scheme of the proposed detection system is given in Section III. The modelling of the spectral and temporal structure of alarms is explained in Sections IV and V, respectively. Section VI describes several post-processing schemes we employed. Finally, in Section VII we provide the information about the evaluation setup and present and discuss the experimental results.

## II. Data Description

**FIGURE 1.**
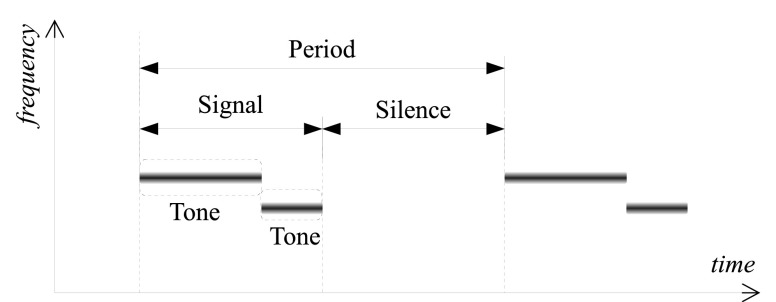
Graphical description of terms used to denote particular alarm properties. Only fundamental frequency is depicted for clarity of presentation [22].

The audio database used in this work contains real-world audio recordings made in the NICU of Hospital Sant Joan de Déu Barcelona during ten recording sessions. Two electret unidirectional microphones connected to a linear PCM recorder were used to make recordings. One microphone was placed inside the incubator, close to the infant’s ear, and the other one outside the incubator, at approximately 50 cm distance above it, usually pointing to the centre of the room. More information about the database acquisition and a general description of the NICU acoustic environment can be found in [21]. The costly manual annotations cover 54.3 min of the audio data and alarm sounds occur 19.28% of this time. Note that each alarm signal (see Figure 1 for notation) was labelled separately. The recordings were downsampled from original 44.1 kHz to 24 kHz.

Observing the audio data collected in the NICU we found 16 different types of acoustic alarms generated by diverse biomedical equipment (cardiorespiratory monitors, infusion pumps, ventilation devices, incubators, etc). From those, 7 types were selected in our tests under the criteria of being the most represented in the annotated data and being relevant from the medical point of view (see Table 1). The general properties of the alarms can be described as:

1) They are periodic in time. Each alarm period consists of signal and silence intervals of established durations (see Figure 1).
2) The signal interval may consist of one or several consecutive stationary signals (tones), each containing one or several simultaneous frequency components, which may or may not be harmonically related.

**TABLE 1:** Detailed Characteristics of the Considered Alarm Classes

Class	Source device	Frequencies (kHz)	Signal duration (s)	Silence duration (s)	Samples
a1	Monitor	i) 0.495, 1.465, 2.435^1^,^2^ ii) 0.515, 2.455, 3.445, 4.415	0.698 ± 0.170	i) 1.352 ii) 1.548	238
a3	Incubator	i) 0.665, 1.330, 1.990, 2.660 / 0.540, 1.60, 3.150^3^ ii) 0.520 / 0.420	0.634 ± 0.142	14.666	130
a6	Ventilator	2.410	0.374 ± 0.069	0.073	203
a7	Monitor	i) 0.980, 2.935 ii) 2.880	0.836 ± 0.188	0.179	114
a8	Monitor	0.490, 1.480, 2.460, 3.440, 4.420	0.280 ± 0.060	1.965	452
a10	Infusion pump	i) 1.140, 2.280, 3.425 ii) 0.880	0.675 ± 0.112	0.325	75
a16	Monitor	0.495	0.307 ± 0.055	1.746	135

^1^ Comma-seperated frequencies are simulations in time.

^2^ Information in each item corresponds to a different version of the alarm.

^3^ Information seperated with slash corresponds to tones consecutive in time.

The particular characteristics of the selected alarm classes are presented in Table 1. The alarm-specific frequencies, signal and silence interval durations were carefully analysed using the recordings made both in the NICU and in a quiet room. The alarm-specific frequency values (with a resolution of 1 Hz) and period durations were obtained by visual inspection of alarm samples. The reported signal interval durations are an average over the annotated samples. Except of one alarm class (a3), all the alarms have a simple “tone-silence” structure. Several alarm classes (namely, a1, a3, a7 and a10) show some variation in the frequency and duration values across device units of the same model. Since for the medical staff such alarms are perceived alike, they are referred to as different versions of the alarm class. According to clinicians, the most important classes are a1, a6 and a7.

Depending on the alarm class, the amount of audio data annotated as belonging to that class was from 1.24 to 5.02% of the total annotated data duration, and the signals of 2, 3 or 4 alarms sounded simultaneously for 6.81%, 0.70% or 0.07% or it, correspondingly.

## III. Overview of the Alarm Detection System

**FIGURE 2.**
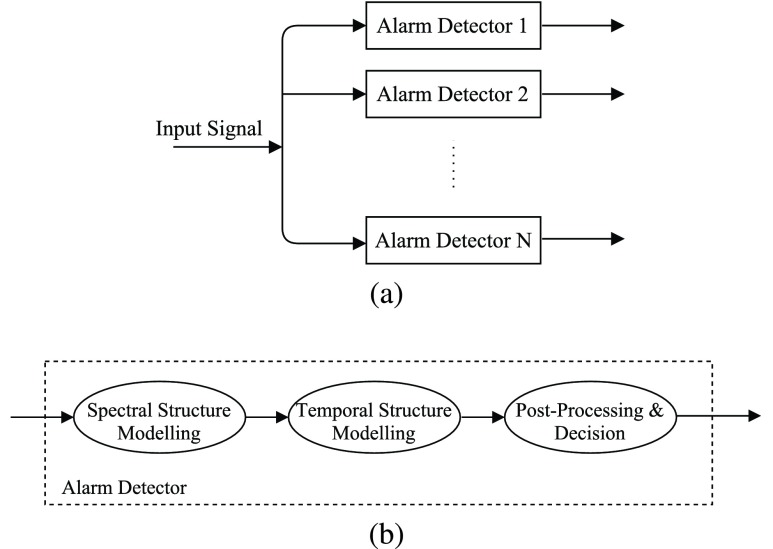
Automatic system for alarm detection: (a) set of parallel detectors, one per class, and (b) diagram of an individual detector.

This section outlines the overall structure of the proposed alarm detection system. The system consists of a set of detectors operating in a parallel manner. Each individual detector is devoted to deal with a particular alarm class and consists of the following blocks: i) modelling of the alarm spectral structure; ii) modelling of the alarm temporal structure; iii) post-processing and decision. The overall structure of the system and of individual detectors is depicted in Figure 2. In an individual alarm detector, the block on modelling of the alarm spectral structure provides probabilities of the presence of the specific type of alarm at the frame level. This block includes feature extraction and statistical modelling stages and the description of the techniques we employed is given in Section IV. The modelling of the alarm temporal structure is based on aggregating the output of class-specific detectors over time and is described in Section V. Finally, the post-processing and decision stages account for assessment at the frame level and at the alarm period level, and their details are provided in Section VI.

## IV. Modelling of the Alarm Spectral Structure

### A. Feature Extraction

We have explored several ways of extracting features from the signal and these are described in the following subsections. All types of feature extraction are performed in the spectral domain. The acoustic signal is split into frames and the discrete Fourier transform (DFT) is applied on each frame. The frame-length of $N=2048$ and frame-shift of $L=1024$ were found a suitable compromise between the time and frequency resolution.

#### 1) Baseline

The features we used as the baseline are referred to as frequency-filtered logarithmic filter-bank energies (FF-LFBEs) [23]. These are generic features used in speech and audio pattern processing. They cover the entire frequency bandwidth. These features were obtained by passing the DFT output through a bank of Mel-scaled triangular band-pass filters [24] and taking the difference between the LFBE of the following and preceeding band-pass filter. We used 18 Mel-scaled filters and the obtained FF-LFBEs were appended with their temporal derivatives, resulting in 36 dimensional feature vector per frame.

#### 2) Sinusoidal Detection Based

This feature extraction scheme is based on the fact that the alarms we are dealing with consist of only sinusoidal components. As such, we employ a method for detection of sinusoidal signals. There have been a variety of methods proposed for detection of sinusoidal signals – for instance, a review of methods used in audio processing is presented in [25]. The method we employed here was introduced in [18]. It considers the sinusoid detection as a pattern recognition problem. We employed this method in our recent research on recognition of bird species from their vocalisa- tions [26], [27] and its earlier form, presented in [28], for analysis of speech [29].

We process each frame $l$ of the signal independently. The peaks in the short-time magnitude spectrum $S_{l}$ are considered as potential sinusoidal components. Let us denote by $k_{p}$ the frequency index of a spectral peak. The peak is characterised by a feature vector $\mathbf {y}=(\mathbf {y}^{1},\mathbf {y}^{2})$, where $\mathbf {y}^{1}$ captures the magnitude shape and $\mathbf {y}^{2}$ the phase continuity and both are formed using $M$ points of the short-time spectrum around the peak. The $\mathbf {y}^{1}$ contains values of the short-time magnitude spectrum around the peak $k_{p}$, each normalised by the magnitude value of the peak, i.e., $\mathbf {y}^{1}=(|S_{l}(k_{p}-M)|/|S_{l}(k_{p})|, \ldots, |S_{l}(k_{p}+M)|/|S_{l}(k_{p})|)$. The $\mathbf {y}^{2}$ is calculated as the difference between the short-time phase of the current $\phi _{l}$ and previous $\phi _{l-1}$ frame, with the shift between the frames being accounted for, i.e., $\mathbf {y}^{2}=(\Delta \phi _{l}(k_{p}-M), \ldots, \Delta \phi _{l}(k_{p}+M))$, where $\Delta \phi _{l}(k) = \phi _{l}(k) - \phi _{l-1}(k) - 2 \pi k_{p} L / N$.

**FIGURE 3.**
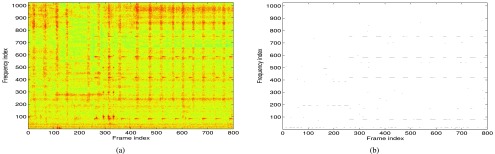
An example of a spectrogram (a) of audio recording and the detected sinusoidal components (b).

A statistical model is built for features representing sinusoidal signals and noise. In this paper, we employed a mixture of multivariate Gaussian distributions to obtain the model for spectral peaks corresponding to sinusoidal signals, denoted by $\lambda _{s}$, and to noise, denoted by $\lambda _{n}$. We found through experimental evaluations the following parameter setup to perform well: rectangular window was used to split the signal into frames, which were then padded with 2048 zeros for the DFT calculation; the parameter $M$ was set to 6 frequency bins; Gaussian mixture models (GMMs) for both the sinusoidal signals and noise consisted of 32 mixture components; sinusoidal signals were corrupted by noise at the signal-to-noise ratio of −7 dB to obtain the sinusoidal model $\lambda _{s}$; temporal segments of a detected sinusoidal component shorter than 3 frames and segments whose average energy was below 40 dB of the maximum average segment energy in a given recording file were discarded. Figure 3 depicts an example of a spectrogram of an audio recording and the detected sinusoidal components. Note that the binary decision about each peak based on the difference $p(\mathbf {y}|\lambda _{s})-p(\mathbf {y}|\lambda _{n})$ is shown. It can be seen that even weak sinusoidal components (e.g. around frequency index 200) are detected well. More thorough evaluations of the detection performance of the method can be found in [18].

In this paper, we form a feature vector consisting of the log-likelihood values $\log p(\mathbf {y}|\lambda _{s})$ and $\log p(\mathbf {y}|\lambda _{n})$ obtained from the sinusoidal detection within the frequency regions around each alarm-specific frequency, as indicated in Table 1, with the tolerance $\delta = \pm 20$ Hz. We further refer to these features as SD LLH (see Table 2). For each alarm frequency region, only the peak that achieves the maximum likelihood on the sinusoidal model is used. A low log-likelihood value is assigned in a case there was no peak in the frequency region. In some experiments, we also incorporate the amplitude structure of the alarms (features SD LLH & Amp). This is performed by including in the feature vector the magnitude values at individual alarm-specific frequency regions. These magnitudes are normalised by the sum of the magnitudes of all the alarm-specific frequencies in order to disregard the effect of the varying alarm amplitude.

**TABLE 2:** Alarm Detection Performance Obtained by a System Modelling the Spectral Structure Only

Features	Dimensionality	Error (MR = FAR) (%)
Baseline	36	35.30
SD LLH ratio	$N_{f}$	32.16
SD LLH	$2N_{f}$	14.52
SD LLH & Amp	$3N_{f}$	13.37
NMF	4	19.41

$N_{f}$ denotes the number of specific frequencies of an alarm class

#### 3) NMF-Based Features

Non-negative Matrix Factorization (NMF) [30] is a linear decomposition technique that attempts to approximate an input non-negative matrix as a product of two non-negative matrices. In audio signal processing, NMF is usually applied to the magnitude spectrogram $S$ of the signal [31], i.e.,

\begin{equation*} S_{N \times T} \approx W_{N \times R} \cdot H_{R \times T}, \end{equation*}

where $N$ and $T$ correspond to the number of frequency bins and number of frames, respectively, and $R \leq N$ controls the rank of the approximation. The columns of $W$ are usually referred to as bases, and the rows of $H$ are their corresponding weights or activations in time.

The problem of minimizing the divergence between the input matrix and its approximation needs to be solved:

\begin{equation*} \arg \min _{W, H} D(S||WH) + \beta \cdot |H|_{1} \quad W,H \geq 0 \end{equation*}

where $D$ is a cost function (in this work, the Kullback-Leibler divergence), and the parameter $\beta \geq 0$ is used to impose a sparsity constraint on the activations. The minimization is achieved by iteratively updating W and H with multiplicative factors (derived using the gradient descent algorithm) until convergence [32].

A supervised NMF approach is used where the bases matrix $W$ is trained beforehand on the training data, and only the activations matrix $H$ is estimated at the source separation step. The bases matrix consists of the bases corresponding to alarm and non-alarm classes $W = [W_{A}; W_{NA}]$. The alarm bases $W_{A}$ are trained for each class separately using the alarm signal intervals and the non-alarm bases $W_{NA}$ are trained using the data segments that do not contain any alarms.

Similarly to works reported in [33] and [34], the feature representation employed in this paper is based on the activations obtained after NMF separation. The activations matrix $H$ is normalised in each frame such that it sums to 1 and only activations corresponding to alarm bases $H_{A}$ are used as features.

In our work, the implementation of NMF described in [32] is used with the following parameter setup: the input matrix $S$ is a magnitude spectrogram computed on Hann-windowed frames. Only the spectral points within frequency regions around each alarm-specific frequency with a tolerance $\delta $ are used for NMF processing. We train $R=4$ and 15 bases per alarm and non-alarm classes, respectively. The sparsity parameter $\beta $ is set to 1. At the training and testing time we use up to 20 iterations.

### B. Statistical Modelling

To perform classification based on the spectral structure features described in the previous subsection we employed both the generative and discriminative approaches, specifically, GMM and Neural Networks (NN).

For each alarm class, a GMM-based detector consists of a model for alarm and a model for non-alarm. Each model is a single Gaussian probability density function with diagonal covariance matrix as, in our experiments, this provided better detection performance than using more mixture components.

The unsupervised pre-training of NN [35] is performed using a Gaussian-Bernoulli RBM [36]. After pre-training, a label layer is added on the top of the network and a supervised backpropagation training is performed, resulting in a discriminative model. Due to the scarcity of data, only networks with one hidden layer are explored in this paper. The hidden and the output layers have 32 and 1 neurons, respectively, and the logistic activation function is used. The training is performed using minibatches, with the size of each minibatch set to 10 and the inputs are randomly distributed among minibatches. The input vectors are mean-variance normalised before being fed to the network; the mean and variance values calculated on the training data are also applied to the testing data. The training data is balanced with regards to classes by randomly selecting samples of the predominant class. The learning rate ($\alpha $), the number of epochs (NoE), the momentum, and weight decay are set, respectively, to 0.001, 80, 0.9, and $2\times 10^{-7}$ during the unsupervised and to 0.001, 50, 0.9, and $1.2\times 10^{-4}$ during the supervised training stage.

## V. Modelling of the Alarm Temporal Structure

In this block, the longer-term time structure of alarms is incorporated as follows. First, for each frame, the log-posteriors of the alarm and the non-alarm class are computed. The logarithm is taken after the probabilities obtained from the statistical models described in Section IV are normalised to sum up to one. Then, these frame-level log-posteriors are aggregated over the intervals corresponding to durations of signal and silence segments in every alarm period. At each frame $t$, the probability of it being the first frame of the alarm period is calculated as

\begin{align} P_{period}(t) = \sum \limits _{i=t}^{t+L_{sig}-1} (P_{A}-P_{NA}) + \sum \limits _{i=t+L_{sig}}^{t+L_{sig}+L_{sil}-1} (P_{NA}-P_{A}) \notag \\ {}\end{align}

where $P_{A}$ and $P_{NA}$ are log-posteriors of the alarm and non-alarm class, $L_{sig}$ and $L_{sil}$ are, respectively, the duration of signal and silence segments in an alarm period.

**FIGURE 4.**
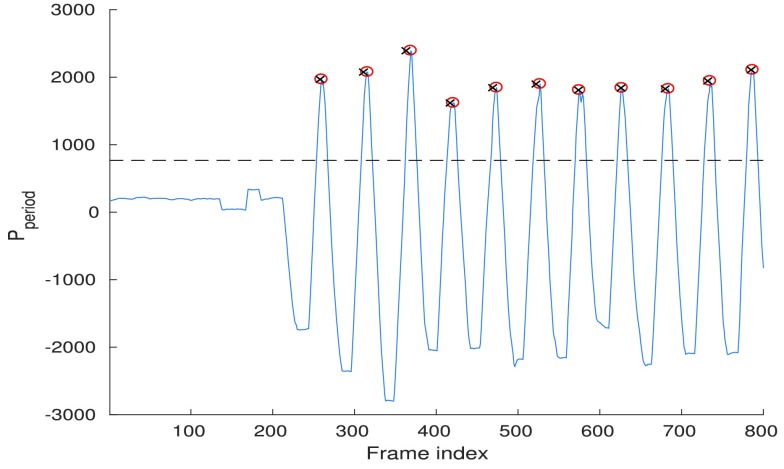
The output of the period probability estimation. Circles correspond to the estimated period timestamps after applying a threshold and crosses are the reference period timestamps.

An illustration of the output obtained from computing that aggregated probability is given in Figure 4. According to the defined expression, each peak of the curve corresponds to the first frame of the estimated alarm period.

## VI. Post-Processing and Decision

Two alternative methods have been considered for the last step in the detection process depicted in Figure 2(b), which include both post-processing and decision.

In the first method, with the likelihoods obtained from the models, each frame is classified either as alarm or non-alarm using the decision threshold that is chosen based on the Equal Error Rate (EER) criterion, so assuming that both miss and false alarm errors are equally important at the frame level. Then the resulting sequence of hypothesized labels is smoothed by means of majority voting. The length of the voting window is set to be the minimum of the signal and silence interval length in an alarm period. For the period-level evaluation, which is described later in Section VII, the beginning of each frame sequence of consecutive alarm labels is regarded as the detected alarm period label. Also, hereafter a constraint of minimal distance between detected periods is applied. That minimal distance is taken equal to 75% of the period duration.

The second method employs the temporal modeling from Section V. The period probability function $P_{period}(t)$ from equation (3) undergoes a class-specific thresholding along the frame index and the peaks of the curve above the threshold are chosen as the detected alarm periods (circles in Figure 4), and are directly used to evaluate at the period level. That class-specific threshold is chosen so as to provide the best period-level performance. To obtain the corresponding frame-level decisions, $L_{sig}$ frames after each of the detected alarm periods are assigned to the alarm class.

A third decision method that results from combining in parallel the previous two methods is also tested in our experiments. In it, if an alarm event (a frame sequence of consecutive alarm labels) detected with the first method does not coincide with any of the alarm periods detected by the second method with a tolerance $\pm L_{sig}/2$, the frames of that event are assigned to the non-alarm class.

## VII. Experimental Evaluation

### A. Evaluation Setup

As the dataset is relatively small, a 10-fold cross-validation scheme was applied to obtain more statistically relevant results. On each data fold, 9 sessions were used for training and 1 session for testing. For each metric, after accumulating the results for each class over all 10 folds, the overall metric scores were obtained by averaging along classes. The same cross-validation scheme was also applied for NMF-based feature extraction, where 9 sessions were used for training the bases, which were applied to process 1 testing session. Only recordings made with the microphone situated outside the incubator were used in the experimental evaluations to keep homogeneous experimental conditions, and also because this microphone is closer to the alarm sources.

During system development, the performance of the detection system was evaluated at the frame level, with two metrics: the Missing Rate (MR) and the False Alarm Rate (FAR), which are defined as

\begin{equation*} MR=\frac {N_{M}}{N_{A}}, \hspace {5mm} FAR=\frac {N_{FA}}{N_{NA}}, \end{equation*}

where $N_{A}$ and $N_{NA}$ respectively are the total number of alarm and non-alarm frames, and $N_{M}$ and $N_{FA}$ are the number of misclassified frames for the alarm class (misses) and for the non-alarm class (false alarms), respectively.

Along with those frame-level metrics, we propose an event-based metric that can offer to clinicians a more meaningful interpretation of the system performance. With that purpose, we chose as event the signal interval inside every alarm period, since it is a naturally perceived acoustic unit. This way, we define the Period-Based ERror Rate (PB-ERR) as a complementary of $F_{1}$-score as

\begin{equation*} \textit {PB-ERR} = 1 - F_{1} = 1 - \frac {2 \cdot N_{C}}{2 \cdot N_{C} + N_{FA} + N_{M}} \end{equation*}

where $N_{C}$ is the number of correctly detected reference alarm periods, $N_{M}$ and $N_{FA}$ are the number of missed and falsely inserted periods, respectively. A reference period is correctly detected if there is a detected alarm period in the tolerance interval $[T_{ref}-T_{tol}; T_{ref}+T_{tol}]$, where $T_{ref}$ is the reference period timestamp and $T_{tol}$ is the tolerance interval duration. Note that $T_{tol}$ must be less than half the alarm period duration; otherwise two reference periods may be associated to one detected period, so counting both of them as correctly detected.

### B. Comparison of Feature Extraction Schemes

First of all, the proposed feature extraction techniques were experimentally evaluated at the frame level, so only GMM modeling of spectral structure was included in the detection system. The post-processing steps were left for subsequent experiments, where GMM modelling is also employed unless explicitly stated otherwise. Results reported in Table 2 correspond to both MR and FAR metrics having the same value (EER).

As it can be seen in Table 2, features based on sinusoidal detection (SD) and on non-negative matrix factorization (NMF), which exploit the knowledge of alarm properties, can significantly outperform the conventional baseline features. The relative improvement obtained is equal to, correspondingly, 62.12% and 45.01%.

The second part of the Table 2 (rows 2-4) shows results when SD is applied for feature extraction. In this case the feature vector can be formed either using the log-likelihood ratio between the sinusoidal model and the noise model (LLH ratio) or using these two log-likelihoods separately (LLH). The performance of the detection system employing the latter features is clearly better as more information is provided to classifiers. To better model the alarm amplitude structure, the LLH features are further combined with the normalized magnitude values (row 4), bringing an additional relative improvement of 7.92%. In fact, the information about the amplitude structure may be helpful for distinguishing between alarms that show very similar frequency components.

The last part of the table presents the results for the NMF-based features and it can be seen that they do not outperform the SD-based features. Actually, their performance is 45.18% relatively worse, which may be explained by the fact that the spectral information captured by NMF-based features is less accurate. In fact, the NMF framework is based on an approximation, which is performed both at the training and the source separation (i.e. feature extraction) steps. While the SD algorithm treats each spectral point independently, in NMF processing, the spectral structure of alarms is captured as a whole by the trained bases. Also, unlike the SD-based features, the activations obtained from NMF processing can be sensitive to the amplitude of the signal.

The alarm occurrences which are most difficult to detect are likely those associated with low SNR values. The effect of such alarm stimuli on the preterm infant is very small, so a more adequate measurement of the detection error may be obtained by discarding the alarm occurrences with low SNR values.

### C. Assessing the Performance of the System According to the Quality of Alarm Samples

In this section we explore the performance of the system considering the quality of the labelled alarms, which is assessed by calculating the local Signal-to-Noise Ratio (SNR) value. The idea is that the effect on the preterm infant of the auditory stimulus due to an alarm is noticeable only if its SNR is sufficiently high. The SNR value is calculated using the recordings made with the microphone placed inside the incubator, so it measures what the preterm infant was receiving. For each alarm sample, the local SNR is calculated around alarm-specific frequency bins $f_{b}$. Both the signal and noise powers are estimated by averaging the spectrum both in frequency (with a margin $\pm \delta $ for the signal and ±100 Hz around the signal margin for the noise) and in time.

**FIGURE 5.**
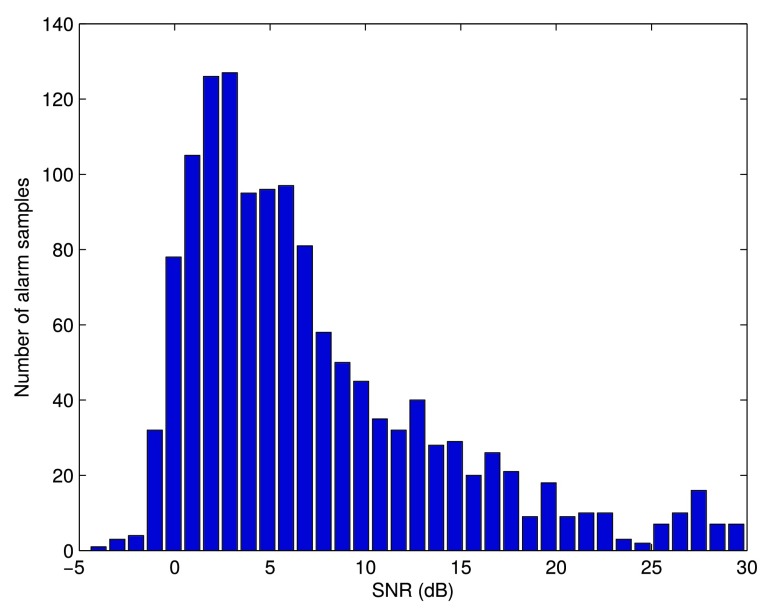
Histogram of SNR values over all labelled alarm samples.

Figure 5 shows the distribution of the alarm samples as a function of their local SNR value. This distribution is exponentially modified Gaussian with the exponential decay towards higher SNR values.

The whole range of SNR values over the labelled database was further divided in 5 dB intervals and all alarm samples were grouped according to these intervals. These groups were evaluated independently and the evaluation results are presented in Table 3 as an average over the considered alarm classes. It can be clearly seen that the system performance improves as the SNR becomes higher and so the quality of the evaluated alarm samples increases. Note that the models used for this evaluation were trained using the whole set of alarms from the database, which means that the models were trained on multiple conditions.

**TABLE 3:** Alarm Detection Performance Obtained Over the SNR Intervals

SNR range (dB)	< 0	0 – 5	5 – 10	10 – 15	15 – 20	20 – 25	> 25	All
MR = FAR (%)	19.79	15.84	13.66	13.19	7.40	9.01	7.27	13.37
Alarms evaluated	77	555	354	122	96	36	47	1347

We further explored how the performance of the detection system changes in case the lowest quality alarm samples are discarded from the evaluation. Table 4 shows the evolution of the detection error with regards to the threshold placed on the SNR values, where alarms with SNR below this threshold are not included in the evaluation. Notice that there is a drop in the detection error when alarm samples with SNR value below 5 dB are discarded, and in that case the detection error (MR = FAR) becomes 10.55%.

**TABLE 4:** Alarm Detection Performance Obtained by Discarding the Alarm Samples Below the SNR Threshold

SNR threshold (dB)	None	0	5
MR = FAR (%)	13.37	13.09	10.55
Alarms discarded	0	77	632

### D. Comparison of Statistical Models

**FIGURE 6.**
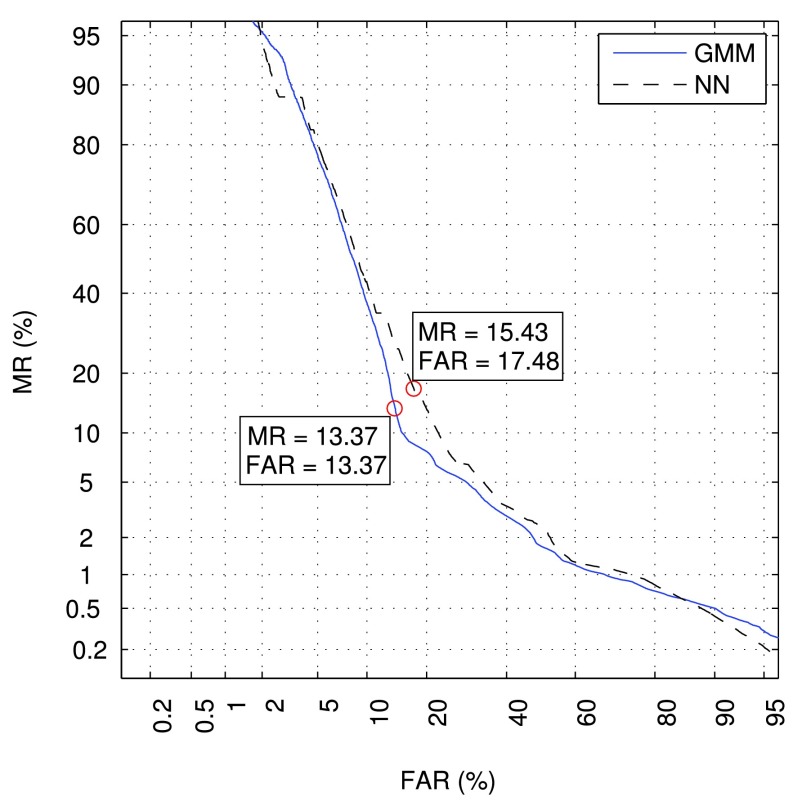
The DET graphs for different statistical models. Circles correspond to points closest to EER.

In this work, we explore two different statistical models described in Section IV. As in the previous subsection, no post-processing schemes are applied, and the best-performing feature extraction setup, namely SD LLH & Amp, is employed. The Detection Error Tradeoff (DET) graphs for the GMM-based and NN-based statistical models are shown on Figure 6. The curves were obtained by varying a threshold on the log-likelihood ratio and averaged over the considered alarm classes. It can be seen that the GMM-based models outperform the NN-based ones at almost all the operating points of the curve, even though the NN-based models are discriminatively trained. This behaviour may be explained by the fact that a very limited amount of data is available for model training, which reduces the generalization capability of the networks and may cause overfitting.

### E. Comparison of Post-Processing Schemes with Application-Specific Evaluation

Table 5 shows the results when post-processing is included before detection in terms of either smoothing (S), temporal modelling (TM) or a combination of both, as described in Section VI. It can be seen that none post-processing scheme improves MR scores compared to not performing any post-processing at all, but all schemes improve the FAR metric scores to a large extent (up to 87.8% relative improvement in the best case). Moreover, all the post-processing schemes are able to improve the PB-ERR scores. Note that for the PB-ERR metric calculation the parameter $T_{tol}$ was set to 49% of the alarm period duration. In fact, according to what is explained in Section VII-A, this is a largest value the $T_{tol}$ can take on. In this case, the system is expected to detect an alarm in the tolerance interval that has the duration of almost one alarm period, which is acceptable for the medical application, taking into account that the period duration of most of the alarm classes is quite short.

**TABLE 5:** Alarm Detection Performance Obtained From Different Post-Processing Methods

Post-processing	Error metrics (%)		
	MR	FAR	PB-ERR
None (baseline)	35.30	35.30	93.45
None	13.37	13.37	68.96
Smoothing (S)	13.70	9.61	53.62
Temp. modelling (TM)	33.56	2.36	36.27
Combination (S & TM)	32.49	1.57	33.10

In general, we could say that smoothing provides better results at the frame level, while temporal modelling performs better at the period level. This fact should be mainly attributed to the way the results are obtained for these post-processing schemes, as described in Section VI. Notice that, although smoothing slightly increases MR, it brings a significant error reduction in terms of FAR(which corresponds to −2.64% and 28.12% relative improvement). Temporal modelling, on the other hand, reduces even stronger the FAR error (by 82.35%, relatively) and is not performing well in terms of MR metric, but gives better period-level score, which is more important for the medical application. Although there is a big difference between frame-level metrics in %, due to the significant unbalance between the alarm and non-alarm classes, in terms of the absolute number of frame errors the deterioration of MR results is smaller than the improvement of FAR results.

The best PB-ERR metric score corresponds to the combination of both smoothing and temporal modelling (S & TM). It is 52% relatively better than not using any post-processing and yields more than 60% absolute improvement compared to the baseline system that uses generic features. The combination of both schemes outperforms the temporal modelling not only in terms of PB-ERR, but also at the frame level. It is worth noticing that in this experiment the system performs 7% relatively worse on segments where several alarms overlap in comparison to segments with no overlaps, which is mainly due to overlaps between alarms that have similar spectro-temporal structure.

**FIGURE 7.**
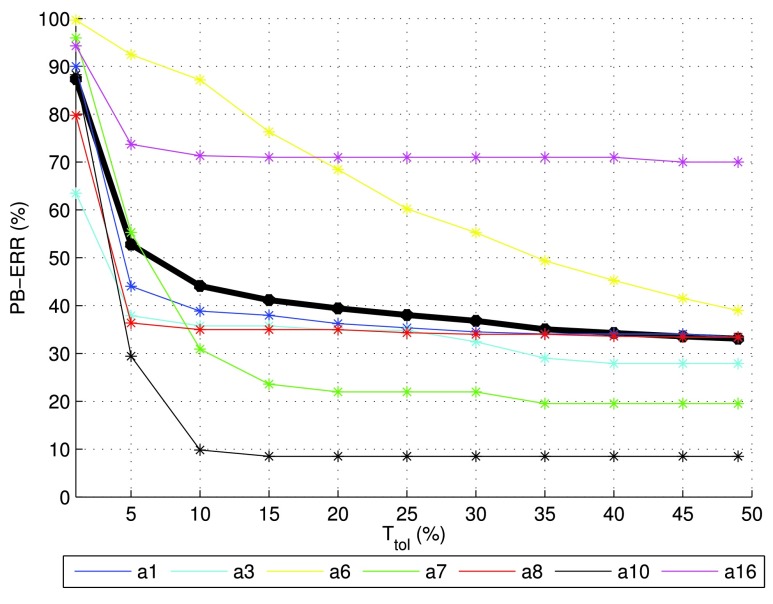
PB-ERR metric result as a function of the tolerance value $T_{tol}$. The bold black line corresponds to the average over the alarm classes. The far right points of the curves are the reported PB-ERR results using 49% tolerance, by classes (%): *a1* 33.33, *a3* 27.90, *a6* 38.99, *a7* 19.51, *a8* 33.50, *a10* 8.50, *a16* 69.98.

Finally, Figure 7 provides more detailed information about the period-level performance for the combined post-processing scheme, and shows the dependency of the results upon the tolerance value $T_{tol}$ used for the PB-ERR calculation. Note from the figure that the performance of most detectors is not improving significantly when $T_{tol} > 15\%$. The best results are obtained for classes a3, a7 and a10, which have spectro-temporal properties quite different from other alarm classes. The worst results, on the other hand, are obtained for the alarm class a16, which shares its only frequency with classes a1 and a8. The results for class a6 are strongly dependent on $T_{tol}$ and a high value is required due to the short period duration of that alarm class.

## VIII. Conclusions

The reported work presents an automatic system for the detection of acoustic alarms in a noisy NICU environment, which is machine learning based but also exploits the knowledge of their particular spectral and temporal properties. In particular, it has been shown that the detection system benefits largely from the introduction of both spectral and temporal information. The experimental results show that the detection errors obtained by the proposed system are still rather high, a fact which may be attributed to the rich multisource, noisy nature of a real-world NICU environment and to the scarcity of the available annotated data.

In order to improve the system performance, some detection hierarchy could be considered, e.g. the alarm classes that have similar spectral structure could be detected consecutively, starting first with those having more frequency components. Also, a more sophisticated algorithm for sinusoidal detection could be employed. Hopefully, the incorporation of much more data will allow high performance improvements.

To be implemented in the hospital environment an enhanced staff notification technology would require two elements: automatic detection of alarm sounds and smart alarm notification. The robust alarm detection system would provide an input event to the alarm notification system, and the latter would infer the clinical relevance of that event based on the severity and urgency of the corresponding alarm, on the occurrence of particular alarm combinations, etc. The overall usability of the notification system would depend on the combination of the above-mentioned elements and would require thorough evaluation of clinical effectiveness. Therefore, future work would entail the development of a complementary smart alarm notification system.
